# Macular hole morphology and measurement using an automated three-dimensional image segmentation algorithm

**DOI:** 10.1136/bmjophth-2019-000404

**Published:** 2020-08-16

**Authors:** Yunzi Chen, Amar V Nasrulloh, Ian Wilson, Caspar Geenen, Maged Habib, Boguslaw Obara, David H W Steel

**Affiliations:** 1 Sunderland Eye Infirmary, Sunderland, UK; 2 Department of Computer Science, Durham University, Durham, Durham, UK; 3 Bioscience Institute, Newcastle University, Newcastle upon Tyne, UK

**Keywords:** macula, imaging, retina, anatomy, diagnostic tests/investigation

## Abstract

**Objective:**

Full-thickness macular holes (MH) are classified principally by size, which is one of the strongest predictors of anatomical and visual success. Using a three-dimensional (3D) automated image processing algorithm, we analysed optical coherence tomography (OCT) images of 104 MH of patients, comparing MH dimensions and morphology with clinician-acquired two-dimensional measurements.

**Methods and Analysis:**

All patients underwent a high-density central horizontal scanning OCT protocol. Two independent clinicians measured the minimum linear diameter (MLD) and maximum base diameter. OCT images were also analysed using an automated 3D segmentation algorithm which produced key parameters including the respective maximum and minimum diameter of the minimum area (MA) of the MH, as well as volume and surface area.

**Results:**

Using the algorithm-derived values, MH were found to have significant asymmetry in all dimensions. The minima of the MA were typically approximately 90° to the horizontal, and differed from their maxima by 55 μm. The minima of the MA differed from the human-measured MLD by a mean of nearly 50 μm, with significant interobserver variability. The resultant differences led to reclassification using the International Vitreomacular Traction Study Group classification in a quarter of the patients (p=0.07).

**Conclusion:**

MH are complex shapes with significant asymmetry in all dimensions. We have shown how 3D automated analysis of MH describes their dimensions more accurately and repeatably than human assessment. This could be used in future studies investigating hole progression and outcome to help guide optimum treatments.

**Key messages**

**What is already known about this subject?**

- Full-thickness macular holes (MH) are classified principally by size, which is one of the strongest predictors of surgical and visual success.

**What are the new findings?**

- MH are complex shapes with significant asymmetry in all dimensions.
- We have shown how three-dimensional (3D) automated analysis of MH describes their dimensions more accurately and repeatably than human assessment.

**How might these results change the focus of research or clinical practice?**

- 3D automated analysis of MH could be used in future studies investigating hole progression and outcome to help guide optimum treatments.

## Introduction

Full-thickness macular holes are a common cause of visual impairment with a prevalence of up to 0.5% in the over 60-year-old age group and are bilateral in 7%–16%.^1 2^ Vitrectomy surgery is an established and successful treatment, with ocriplasmin and expansile gas also effective in a lower proportion of selected patients.^3 4^ Macular holes are classified partly by the presence of vitreoretinal adhesion at the fovea and optic disc, but principally by their size.^5^ Indeed, size is used to guide the choice of treatment and the optimum surgical approach, and to predict outcome. A variety of size measures have been described, with minimum linear diameter (MLD) being used to divide holes into small, medium and large.^5^ Ratios of various size parameters have also been suggested, including diameter hole index, macular hole index and macular hole closure index.^6–9^ Similarly, the difference between base diameter (BD) and MLD has been shown to predict response to ocriplasmin.^10^ All these measures have typically been made using a single two-dimensional (2D) slice of a horizontal optical coherence tomography (OCT) image, and measured by a human grader using callipers. This is known to be prone to high intraobserver and interobserver error and also vulnerable to further error from off-centre scan location.^11^ Furthermore, the true three-dimensional (3D) measures of the macular hole are not measured, with symmetry in the X/Y axis being assumed.

We have designed a 3D automated image processing algorithm which is able to segment macular holes with high accuracy. We describe the dimensions and morphology of a consecutive cohort of 104 macular holes from patients prior to surgery and compare them with clinician-acquired measurements in 2D.

## Methods

The spectral domain OCT images of a consecutive cohort of patients assessed for vitreoretinal surgery for idiopathic primary full-thickness macular hole over a 2-year-period in a single eye hospital were prospectively collected as part of routine care and were retrospectively analysed. Secondary, myopic, fellow and persistent holes after previous surgery were all excluded, as were eyes with axial lengths of less than 22 mm and greater than 25.5 mm. All had undergone spectral domain optical coherence tomography (SDOCT) imaging using the Heidelberg Spectralis (Heidelberg, Germany) as part of routine care, using the same imaging protocol. A high-density central horizontal scanning protocol with 29–30 μm line spacing was used in the central 15 by 5 degrees. The individual OCT line scans were 768 by 496 pixels, with the scaling varying slightly between data sets but typically equating to 5.47 μm per pixel in the X (horizontal) axis and 3.87 μm per pixel in the Y (vertical) axis. With 29–30 μm spacing between scans (Z axis), there were 49 scans per data set. All scans used a 16 automatic real-time setting enabling multisampling and noise reduction over 16 images. Prior to image export, two independent experienced clinicians measured the MLD and BD. Observer 2 also measured hole height as previously described and the height above the inner surface of the retinal pigment epithelium (RPE) at which the MLD was measured. MLD was defined as the horizontal minimum hole diameter in the approximate mid-zone of the hole away from any operculum, in the OCT slice with the widest dimensions.

The presence of any vitreomacular traction (VMT) was noted. In the case of any of the measurements being greater than 15% different between the observers’ measurements, the two observers were asked to independently check their measurements to ensure no transcription errors had occurred. The volume of the hole was calculated using the volume of a truncated cone formula as previously used.^12^

Each person’s image data set, comprising approximately 49 scans, was exported as a folder of anonymised non-compressed.tiff files with the image information including the X and Y axes pixel to micron conversion ratio.

The data sets were then analysed using an automated 3D segmentation algorithm as previously described. The system uses a state-of-the-art level set method based on the local Gaussian distribution fitting energy functional, employing a 3D multiscale approach. This is followed by a novel curvature-based surface cutting procedure, which separates the macular hole from its background, allowing for fully automatic measurement of the shape and volume.^13^ We have previously shown that the method is stable to a variety of different macular hole shapes and more accurate than other existing graph cuts segmentation approaches, with an accuracy of segmentation of 99.19% as compared with a ground truth manual segmentation approach by an experienced clinician. The procedure is also highly repeatable.^13^

**Figure 1:**
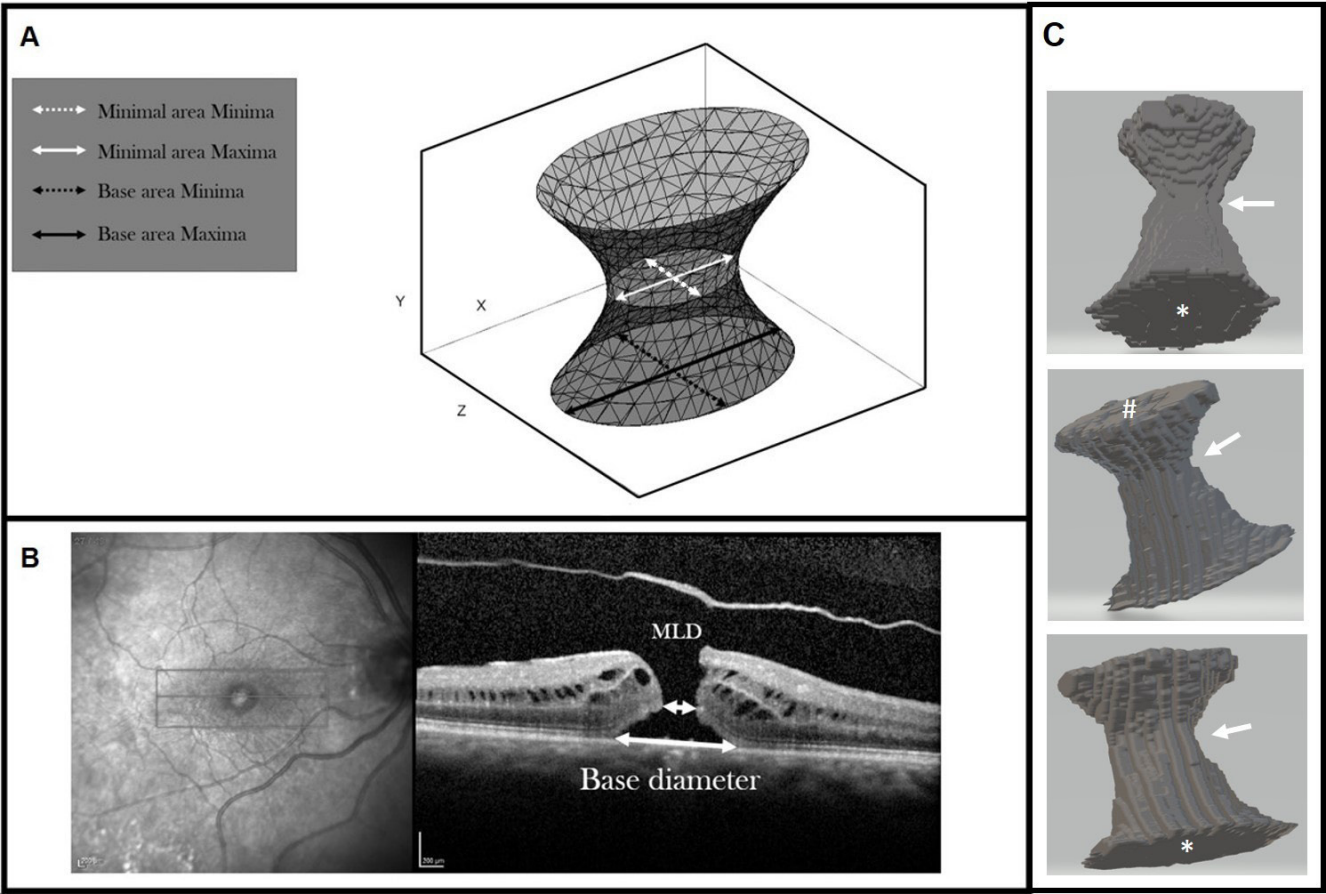
A. Schematic diagram of the macula hole 3D model; B. OCT of a macula hole with 2D labels; C. Representative example of a segmented 3D macular hole in 3 different orientations – base area marked by *, top area (i.e. at the ILM side) marked by #. The approximate zone of the minimal area is shown by the arrow.

A 3D model of the macular hole was produced with the following axes (figure 1):

- X: the axis along the base of the macular hole in the horizontal line scan.
- Y: the axis representing the vertical retinal height, from the RPE to the internal limiting membrane (ILM).
- Z: the axis across the macular hole slices and at right angles to X and Y.

The following parameters were derived from the 3D model and expressed in micron units:

- The maximum and minimum diameters of the base area (BA) of the hole, that is, the area of the hole in the plane of the RPE, and the axes of the maxima in the XZ planes of the scan. The maximum diameter most closely represents the BD measured clinically.
- The maximum and minimum diameters of the minimum area (MA) of the hole defined as the minimum area in the central 20%–90% of the hole height. The minima of the MA most closely represent the MLD as used clinically and in the international VMT classification. The meridian of the minimum axis in the XZ planes was also measured, as well as the height of the minimum area as measured perpendicularly above the RPE.
- The total surface area (SA), defined as the total surface area of the extracted 3D macular hole shape including both base and top areas, and the volume of the extracted 3D macular hole shape measured in pixel areas and voxels, respectively.

Patients or the public were not involved in the design, or conduct, or reporting, or dissemination of our research. Preoperative visual acuities were recorded using Early Treatment Diabetic Retinopathy Study (ETDRS) letter charts and converted to logMAR (logarithm of the minimum angle of resolution) visual acuity for analysis.

### Statistical analysis

Descriptive and statistical analyses were performed using R^14^ and plots using ggplot2.^14 15^

Macular hole variables are presented in terms of mean, with the five quantiles of the distribution given, and percentage as appropriate. Distribution plots are given for a variety of parameters.

Association between continuous data was assessed using Spearman’s rank correlations and between categorical data using two sample t-tests. Stepwise multiple regression was used to analyse the effect of multiple variables. Statistical significance was considered with a p value of 0.05 or less.

## Results

Image data sets and clinical data on 104 eyes from 104 patients were analysed. The mean age was 70 years old (SD 6.6, range 48–84), 85 (82%) were female and 52 (50%) were right eyes. VMT was present in 27 (26%).

### 3D image analysis

The parameters as measured by the image analysis approach are presented in table 1. The mean diameter of the MA was 384.7 μm. There was a mean difference of 54.87 μm between the maximal and minimal dimensions of the MA and 87.14 μm for the BA, representing 17% and 12% of the mean dimensions, but ranging up to 48% and 36%, respectively.

**Table 1:** Baseline parameters derived from image analysis algorithm

	Mean	Minimum	25th percentile	Median	75th percentile	Maximum	P value
Retinal height at centre of hole (μm)	366	223	333	365	396	488	0.78
Minimum dimension of MA (μm)	357	110	247	353	440	871	0.00017
Maximum dimension of MA (μm)	412	147	302	381	490	927	0.0031
Height of centre of MA above RPE (μm)	189	50.3	136	200	229	342	0.43
Difference between maxima and minima of MA (μm)	54.9	4.71	30.8	48.8	76.1	144	0.0011
Minima of BA (μm)	716	148	585	727	876	1410	0.86
Maxima of BA (μm)	803	193	636	823	972	1470	0.68
Difference between maxima and minima of BA (μm)	87.1	15.3	54.2	79.7	108	267	0.00011
Surface area (mm^2^)	1.66	0.254	1.16	1.51	2.07	4.28	0.00022
Volume (×10^−3^ mm^3^)	0.74	0.07	0.43	0.65	0.95	2.36	7.4e-07

P value refers to the Shapiro-Wilk test for normality for each variable. Values less than 0.05 signify that the values are not normally distributed.

BA, base area; MA, minimum area; RPE, retinal pigment epithelium.

There was no clear trend for these meridian differences to vary in extent in holes of different sizes (online supplementary figure 1).

10.1136/bmjophth-2019-000404.supp1 Supplementary data

The mean angle to the X axis of the minimal dimension of the MA was approximately 90° in the XZ plane, that is, at nearly right angles to the horizontally acquired OCT scan. Only 10 of the 104 (10%) image data sets had a minimal dimension within 10° of the horizontal (online supplementary figure 2).

By distinction, the maximum dimension of the BA was more typically nearer the X axis, with 40 of the 104 image data sets (38%) having a maximum base dimension within 10° of the horizontal.

**Figure 2:**
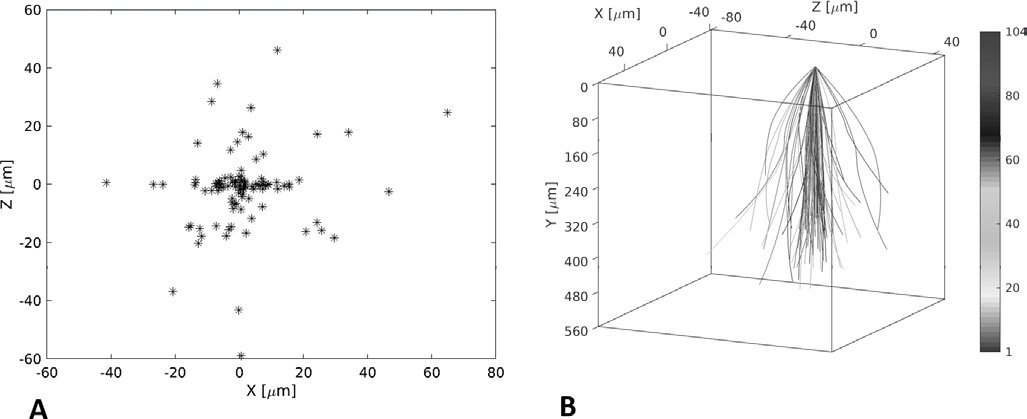
Mapping of centre point of the hole to the base area (A) and the centre line of the hole at the apex compared with the retinal surface (B).

To assess the vertical symmetry of the macular hole, the centre point of the hole on the inner surface was mapped to the BA (figure 2A), with the centre line of the holes shown schematically in figure 2B, showing that several of the holes did not have an orientation perpendicular to the retinal surface.

### Relationship between measured parameters

**Figure 3:**
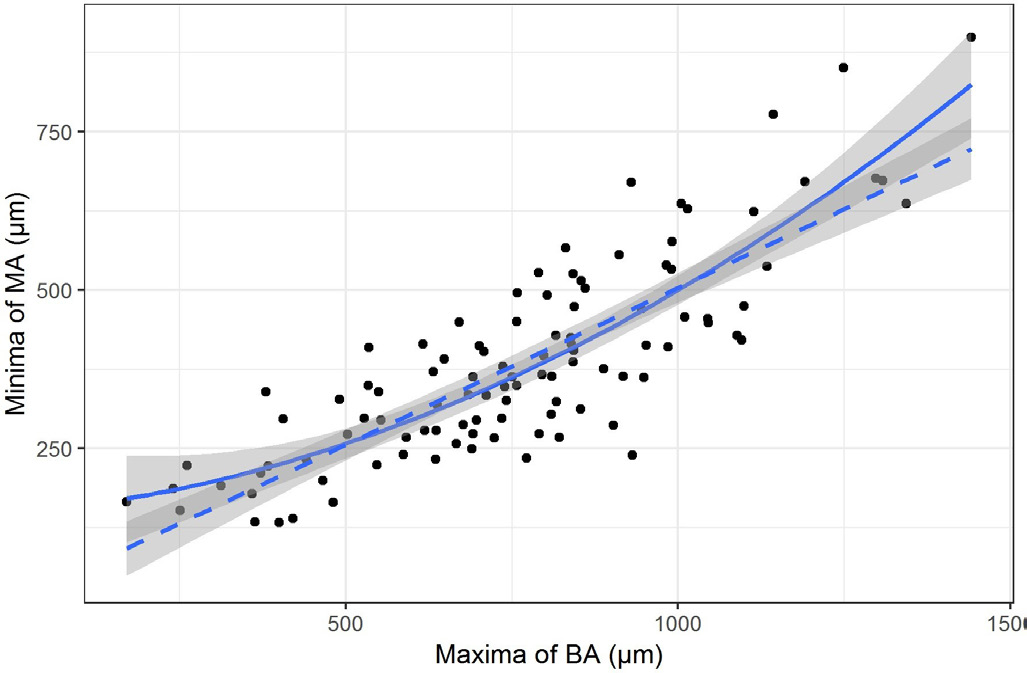
The relationship between the mean dimension of base area (BA) and the mean dimension of minimum area (MA).

The minimum dimensions of the MA were related to the maximum of BA with a quadratic relationship. The rate of increase of the maximum BA was smaller relative to the rate of increase of the minima of the MA particularly for larger holes. The fit of the quadratic (shown as solid line) was better than the linear fit (shown as dotted line with slope beta=0.5, p=0.004) (figure 3).

The height of the MA increased with the width of the MA. Wider holes had MAs that were higher from the RPE than narrower ones (online supplementary figure 3). Several of the macular hole dimensions were highly collinear, in particular the MA, BA, SA and volume. Retinal height, however, was weakly correlated with BA, volume and SA (online supplementary figure 4).

### Macular hole shape and relationship between VMT and hole size

**Figure 4:**
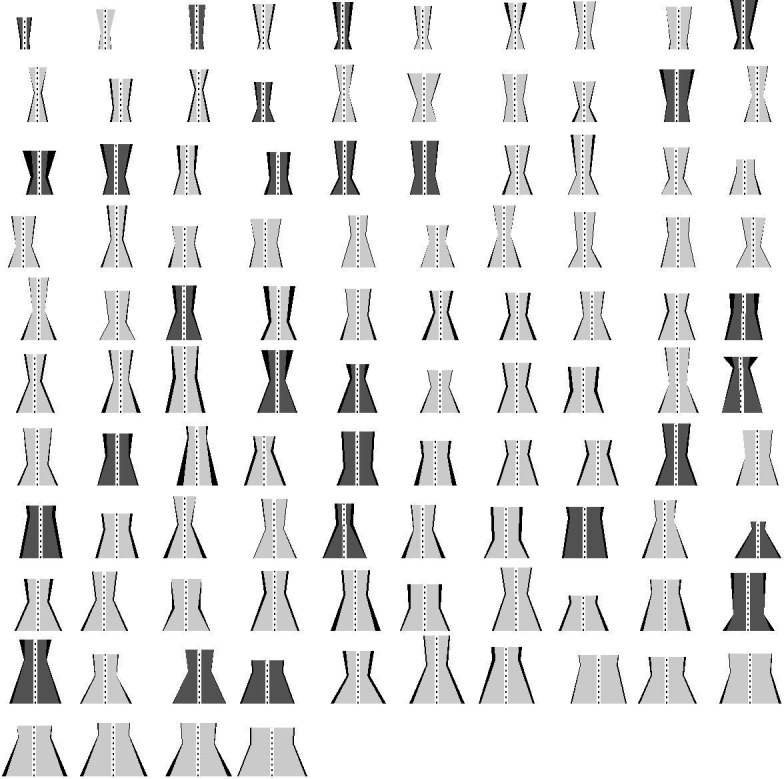
Schematic diagrams of the extracted three-dimensional macular hole shapes using the following dimensions: mean of the base area, mean of the minimum area, height above the base of the minimal area, mean of the top area, and height of the hole from the retinal pigment epithelium to the inner retinal surface. The asymmetry in maximum and minimum dimensions is represented in the thickness of the sides of the schematic holes.

Hole shape varied widely as shown schematically in figure 4, where the holes are ordered according to BA. The presence of VMT (shown as darker shading) was not related to hole size.

### Human measurements and their relationships to algorithm values

The measured values for the macular holes for observers 1 and 2 are shown in online supplementary table 1. There was no significant difference for MLD between observers 1 and 2 (p=0.69), but there was a significant difference in measured BD between the two observers (mean of the differences 38.75 μm, with observer 2 overestimating BD compared with observer 1; p<0.0001) (see online supplementary figure 5). The 95% limits of agreement between the two observers for MLD were −140.3 (−164.6, –115.9) to 145.9 (121.6, 170.3), and those for BD were −161 (−181.8, –140.2) to 83.5 (62.7, 104.3).

**Figure 5:**
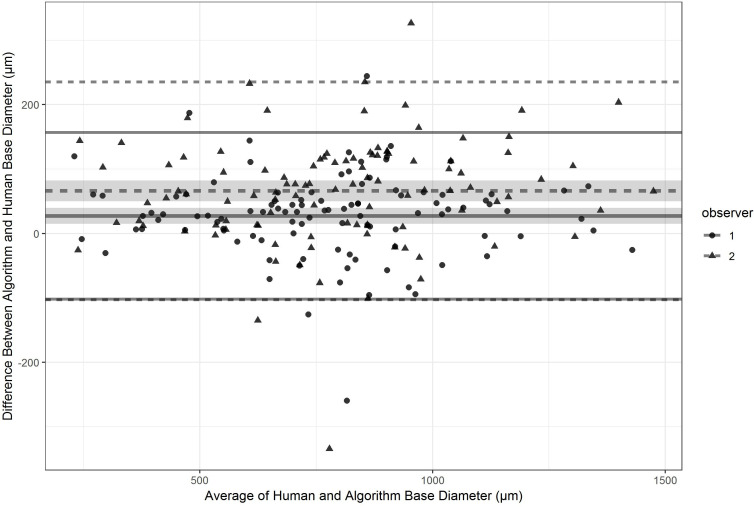
Bland-Altman plots for base diameter measurements with observer 1 against the algorithm and observer 2 against the algorithm superimposed. 95% CIs are shown for the mean differences (shaded) and 95% CIs for the differences (lines).

The mean of both observers for MLD and BD differed significantly from the algorithm-acquired measurements (minor axis of the minimal area and major axis of the BA; p<0.0001 for both), with both observers overestimating MLD and BD significantly (figure 5 shows the results for BD). However, when the mean of the human measures (mean 405 μm) was compared with the equivalent measurement computed by the algorithm (eg, the horizontal dimension of the minimal area, mean 388 μm), the measurements were not significantly different (p=0.46).

The MLD height was not significantly different from the algorithm-derived values (mean 189.1 vs 190.5; p=0.76), but as can be seen in online supplementary figure 6 there was wide variability between the values.

The retinal height was significantly different from the algorithm-acquired measurement (mean 366.3 algorithm vs 389.6 human; p<0.001).

Using the human-measured MLD, 16 holes were classified as small (<250 μm), 44 medium (251–400 μm) and 44 large (>400 μm). Using the algorithm measures of the minimum dimension of the MA as compared with the mean of the human-measured MLD resulted in a change in classification in 25 of the 104 eyes, with 26 classified as small, 48 medium and 30 large (p=0.07) (online supplementary table 2).

The human-derived volume differed significantly from the algorithm value with high variability. Volume was underestimated using the human observers’ measurements, with a trend to increased variability as hole size increased (online supplementary figure 7).

### Associations with preoperative variables

Age, gender, VMT presence and laterality were not associated with any size variable. Preoperative visual acuity was positively associated with size both for MA (r^2^=0.57, higher for minimum than maximum diameter) and BA (r^2^=0.56, higher for maximum diameter than minimum), as well as volume (r^2^=0.54), SA (r^2^=0.57) and height of the MA (r^2^=0.45), but not retinal height (r^2^=0.08) (online supplementary figure 8). Using the mean human measurements, the correlation values were slightly lower at MLD (0.49) and BD (0.48).

## Discussion

We have described the 3D morphology of macular hole using a novel and validated automated 3D segmentation algorithm. The algorithm is robust and was able to accurately segment the full consecutive series of 104 OCTs included in the study, including when there was VMT present. We used a high-density scanning protocol with 30-μm line spacing and averaging 16 A scans per line, reducing noise and meaning that the scan lines were more likely to include the maximum hole dimensions.^11^ We have previously shown that the 3D methodology can very accurately segment out the macular hole boundaries as compared with a human observer, and can therefore be regarded as providing a ground truth for macular hole dimensions and shape. Macular holes are shown to be complex shapes with significant asymmetry, meaning that conventionally acquired clinician measurements fail to represent their key parameters accurately. For example, we found that the XZ meridian of the minima of the MA was only within 10° of the conventionally measured horizontal X axis in 10% of cases, and differed from the human-measured MLD by a mean of nearly 50 μm and up to 200 μm. Similarly, the true maximum BD varied from the mean of the human measurements by 87 μm or 12%. The resultant differences led to a reclassification in size using the International Vitreomacular Traction Study Group classification in a quarter of the patients.^5 16 17^ This has significant implications for studies using macular hole measurements to predict outcomes and to act as cut-off points for deciding on treatments.

The human measurements had a consistent tendency to overestimate the widths of the holes. To measure a macular hole MLD, a human observer must first accurately locate the scan line with the greatest dimensions and then pick the minimum hole dimension, avoiding the area of the operculum if present. The minimal dimension is typically measured parallel to the RPE. Measuring macular hole using a horizontal line scanning protocol relies on the macular hole being symmetric, but we show that the holes were significantly asymmetric in all dimensions. There was a mean difference of 55 μm in maximum and minimum dimensions of the MA and 87 μm for the same measures of the BA. These differences concur with those found by Philippakis *et al*^17^ using en face SDOCTs to measure macular hole dimensions, although they did not comment on the orientation of the maximum/minimum measurements. The minima of the MA were typically approximately 90° to the horizontal, while the maximum of the BA was predominantly horizontal. The holes were therefore oval with their maximum dimension in the XZ axis at the horizontal meridian. Interestingly this corresponds to asymmetries found in the foveal avascular zone (FAZ), where previous studies have found an approximate 30-μm difference, with the horizontal diameter being widest.^18^ It is known that FAZ size is closely related to foveal floor size, and a recent study has suggested an association between macular hole size and foveal floor width.^19 20^ The clinically acquired measurement of MLD is thus typically measured in an axis that does not coincide to its true minimal dimension. Indeed, using an algorithm-derived horizontal dimension of the minimal area, corresponding more to the human MLD measurement, there was no significant difference in the measurements.

Although the holes were generally vertical, the centre point of the MA and BA was misaligned by over 150 μm in 70% of the eyes. This therefore adds to the measurement error of human graders who have tended to measure the MLD and maximum BD on the same SDOCT slice when in reality this occurrence will rarely occur. These asymmetries further explain the human measurement error compared with the true measurements found by the algorithm, as well as interobserver variability. The 95% limits of agreement between the two observers for MLD were −140 to 146, which is in broad agreement with the values found by Banerjee *et al*.^21^ We asked observer 2 to record the height above the RPE at which they measured the MLD, and although it was not significantly different from the height the algorithm measured the minimal area at, it varied from the algorithm by more than 40 μm in 29% of eyes, which is likely another source of error. The two observers were both experienced in measuring macular holes and from the same institution, and it is likely that less experienced observers, with different training, may have had even greater differences between them. If different scanning protocols and OCT machines were added, then the differences would be greater again. We did not assess intravisit variability, which would have increased the variability further.

It is thought that most macular holes are formed by the effects of anteroposterior vitreoretinal traction and VMT. Of the holes in this series 26% had VMT, which is in keeping with previous figures from the same population area.^22^ We found no significant association between the presence of VMT and any of the size parameters measured, which is in keeping with the findings of Philippakis *et al*.^23^

We found that age and gender were not significantly associated with the algorithm and human-measured values, but preoperative vision was, as other authors have found.^24 25^ The strongest relationships were all those derived from the algorithm as opposed to the human observers. However, the preoperative visions were checked without a protocol refraction and exact relationships are uncertain.

Three other approaches have been suggested to evaluate macular hole shape and dimensions beyond human measurements from standard OCT line scans. Philippakis *et al*^17^ elegantly demonstrated the use of en face reconstruction to measure macular hole minimal area and dimensions. The technique however had a high technical failure rate of ~50%, often had to be manually adjusted when VMT was present and was unable to measure other hole parameters. Problems may also be encountered where holes are misaligned vertically as we have already observed above. Geng *et al*^26^ used a manual segmentation technique combined with Matlab to produce a 3D representation of the hole from which 3D parameters could be measured, but involves a time-consuming manual mark-up. Xu *et al*^27^ have described an approach of automatically measuring macular hole dimensions based on the sum of 2D images. In comparison, our algorithm considers the overall 3D geometry of the hole and is significantly faster. We have also validated the accuracy of our system against human segmentation in a set of 30 eyes and showed very high accuracy.

Our study has several limitations. We did not correct the measurements for axial length, although we restricted the entry criteria to eyes with axial lengths between 22 and 25 mm. Furthermore, inaccuracies introduced by doing this would only affect absolute measurements, not the differences in dimension we describe nor differences from the human measures. We used a specified scanning protocol by one OCT manufacturer, which also limits the applicability of our technique and interpretation of our findings. Similarly, although a consecutive cohort, our sample was restricted to patients undergoing surgery in one centre, which may not be representative of all idiopathic macular holes or other ethnicities and populations.

In conclusion we have previously described a 3D automated macular hole segmentation system that is able to accurately segment out a macular hole from its constituent cross-sectional 2D scans. We now present the detailed findings from a cohort of 104 consecutive macular holes, with description of several clinically relevant 2D and 3D dimensions derived from the 3D macular hole shape extracted. We show that the measurements are significantly different from those measured by experienced human graders. Macular hole size is known to be one of the strongest predictors of surgical success both anatomically and functionally. Evaluation of the measurements generated from this automated system in a prospectively collected data set of eyes undergoing surgery with outcomes analysis will be of great interest.
