# Supplementary material for: Macular hole morphology and measurement using an automated three-dimensional image segmentation algorithm
Source: BMJ Open Ophthalmol. 2020 Aug 16;5(1):e000404. doi: 10.1136/bmjophth-2019-000404 (PMC7430427; doi:10.1136/bmjophth-2019-000404)
Supplement: Supplementary data [file bmjophth-2019-000404supp001.pdf]

Supplementary material

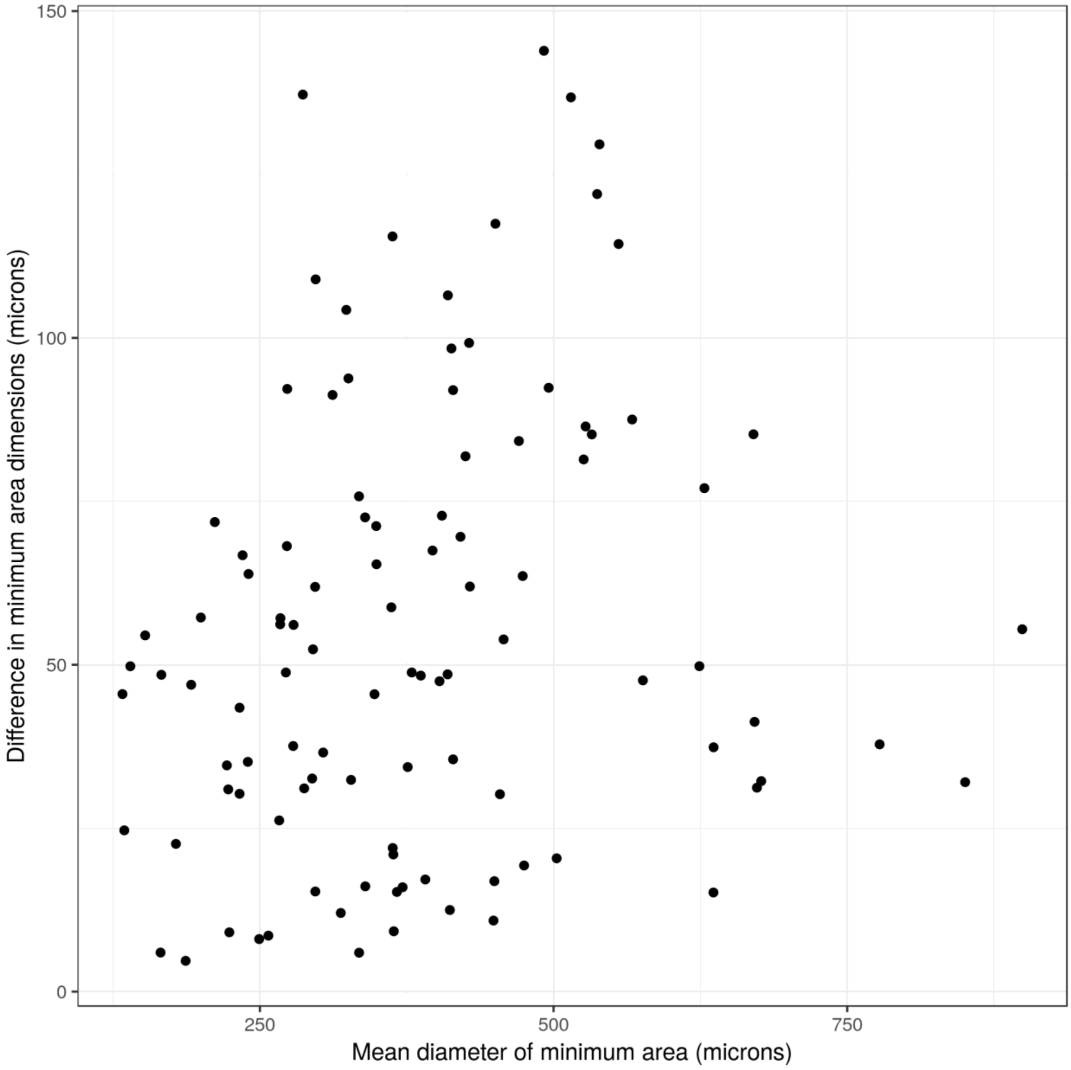

Supplementary figure 1: Difference in the maximal and minimal dimensions of the MA compare to the mean diameter of the MA.

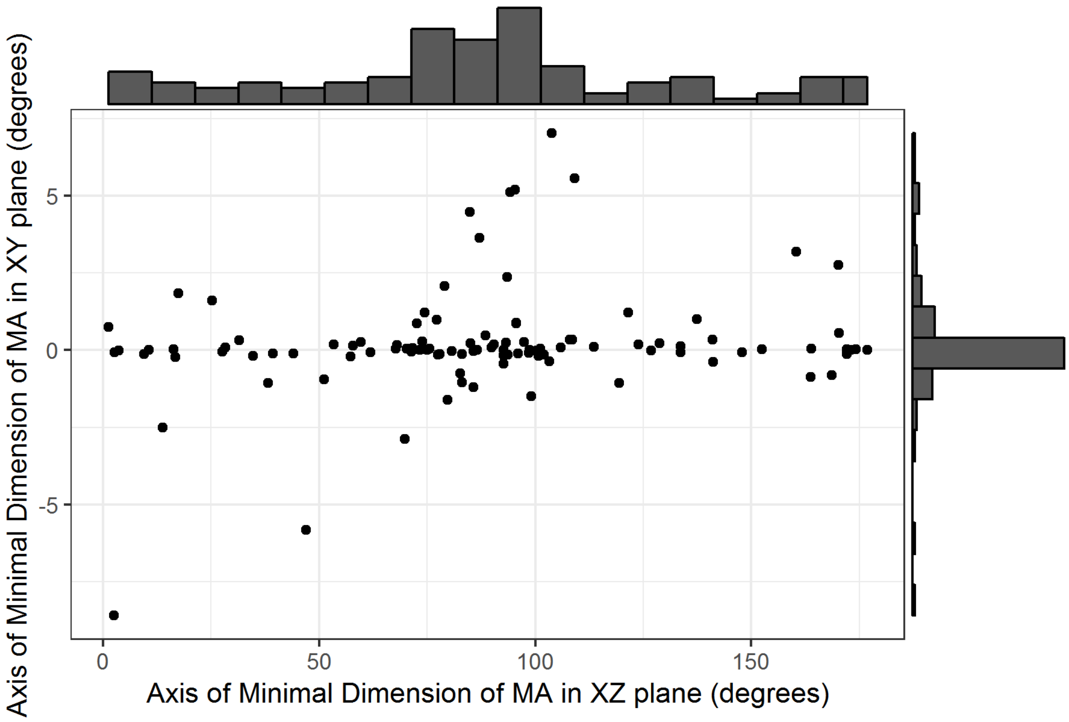

Supplementary figure 2: Axis of minima of minimum area in XZ plane compare to its axis in XY plane.

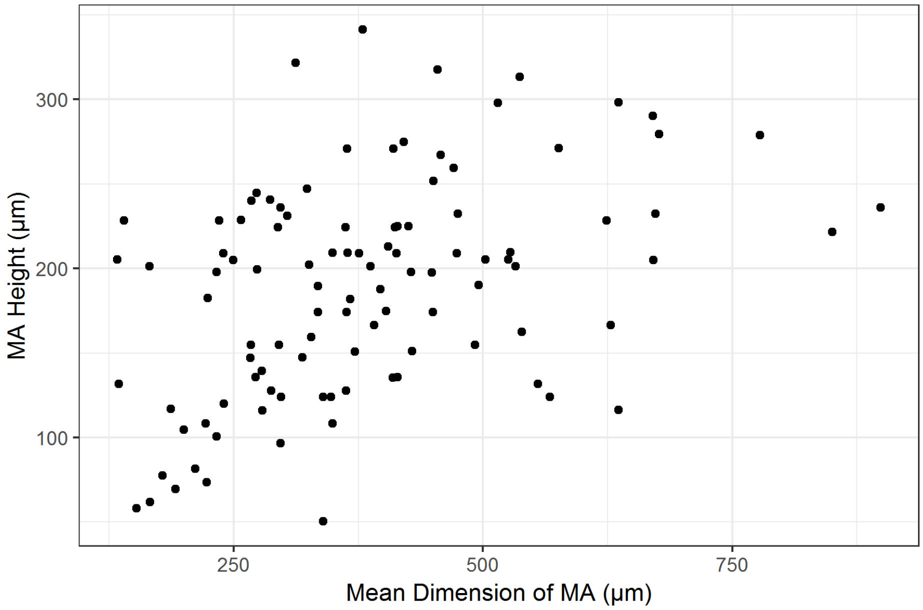

Supplementary figure 3: Mean width of MA compared to the height of MA.

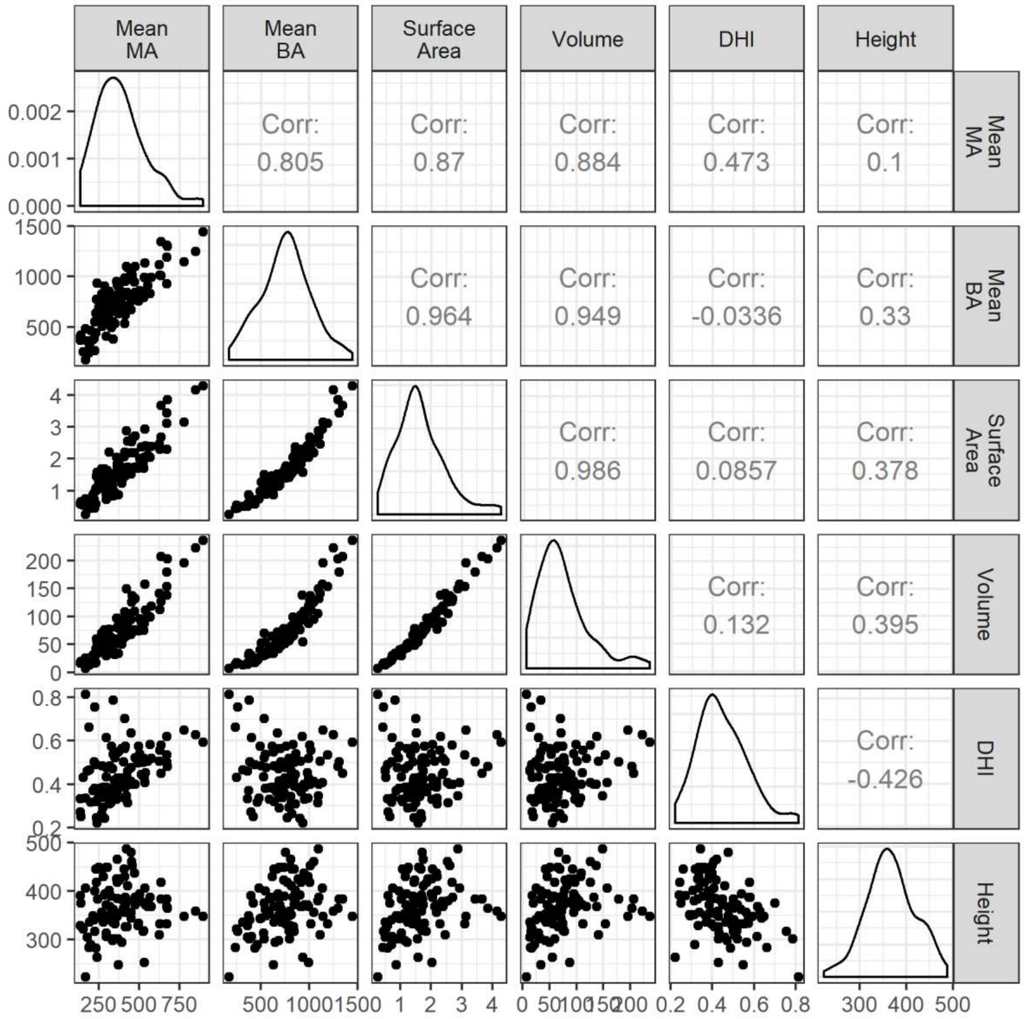

Supplementary figure 4: Correlation between mean dimension of MA and BA, surface area, volume, DHI and height.

Supplementary table 1: Observers 1 and 2 macula hole measurements based on 2D OCT images

Observer	Parameter	Mean	SD	Minimum	Maximum
1	MLD (microns)	406.6	151.9	122	885
	BD (microns)	787.1	255.1	242	1416
2	MLD (microns)	403.8	179.5	105	1122
	BD (microns)	825.8	268.1	225	1507
	Hole height (microns)	389.6	55.2	240	515
	MLD height (microns)	190.5	55.8	71	341
Mean of observer 1&2	MLD	405.3	162.2	113.5	965
	BD	806.5	259.9	233.5	1461.5
Difference between mean of observer 1 & 2 versus algorithm derived values	MLD (microns) (% difference from algorithm)	+47.9 (11.9)	53.4 (13.3)	-90.8 (-26.9)	212.8 (49.2)
	BD (microns) (% difference from algorithm)	+87.1 (12.2)	47.4 (6.6)	15.3 (1.79)	266.7 (36.2)

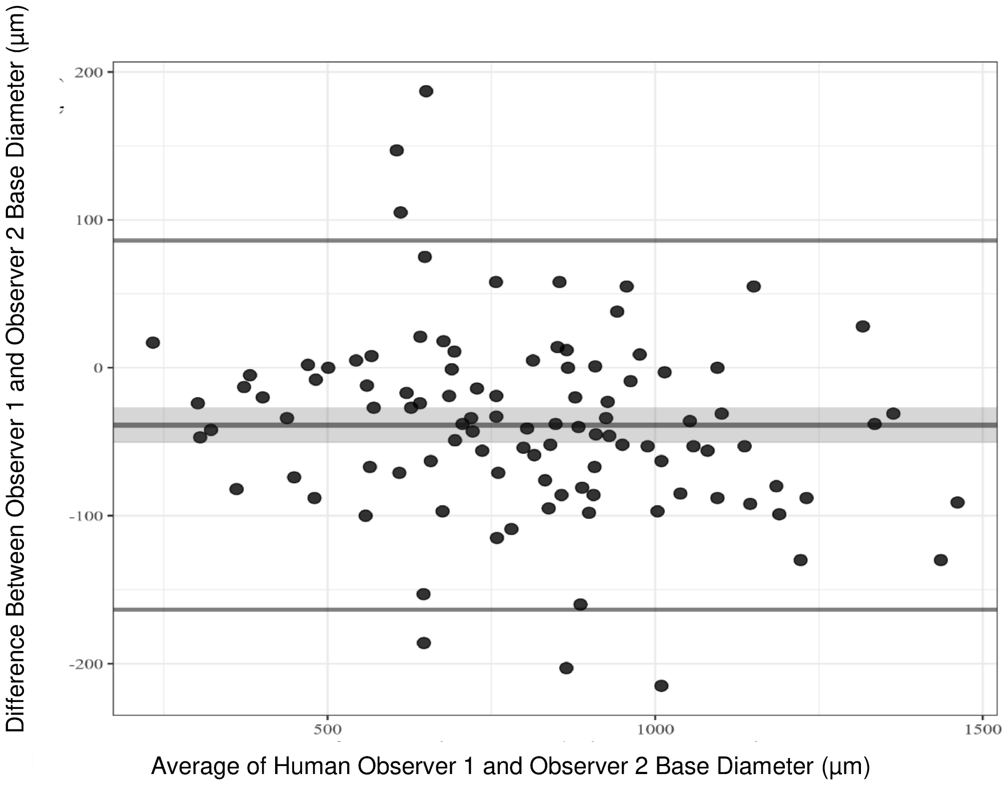

Supplementary figure 5: Bland Altman plots for human observer 1 and observe 2 measured base diameter. The 95% confidence intervals are shown for the mean differences (shaded) and 95% intervals for the differences (lines).

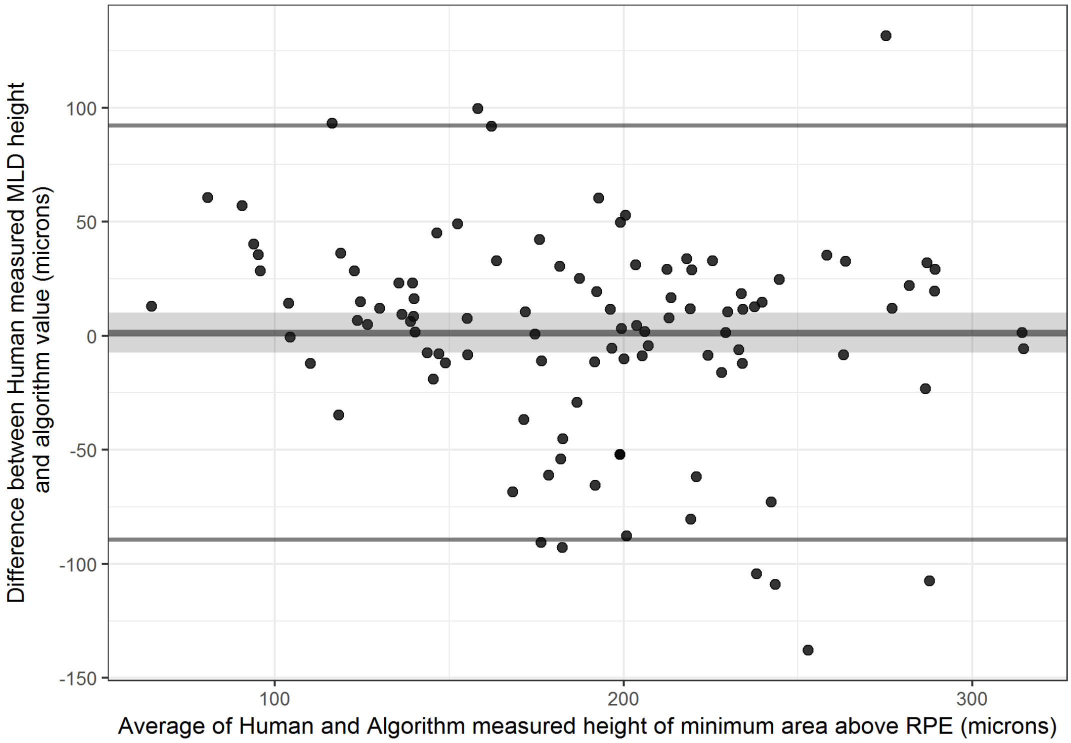

Supplementary figure 6: Bland Altman plots for algorithm derived measured height of minimum area above RPE and human measurements of MLD height. The 95% confidence intervals are shown for the mean differences (shaded) and 95% intervals for the differences (lines).

Supplementary table 2: classification of MH based on size. Algorithm derived measurements in black, human measurements in grey.

	<250	***250-400***	***>400***	**Algorithm**
<250 microns	16	9	1	26
250 - 400 microns		33	15	48
>400 microns		2	28	30
**Human**	16	44	44	104

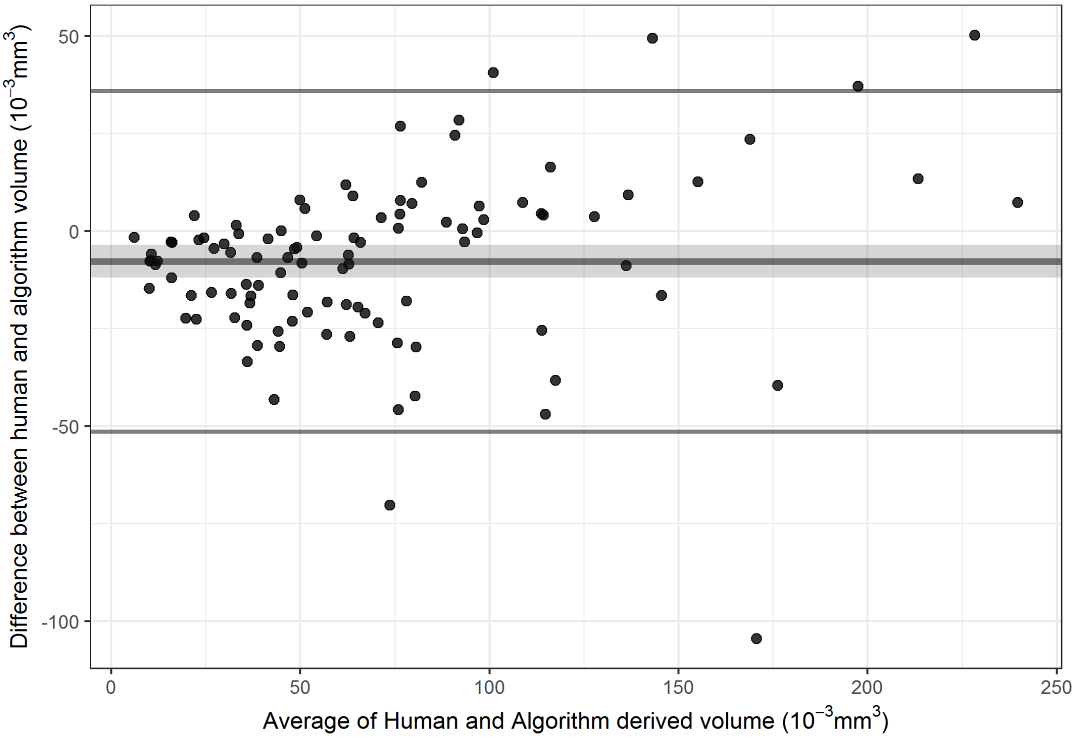

Supplementary figure 7: Bland Altman plots for human and algorithm derived MH volume. The 95% confidence intervals are shown for the mean differences (shaded) and 95% intervals for the differences (lines).

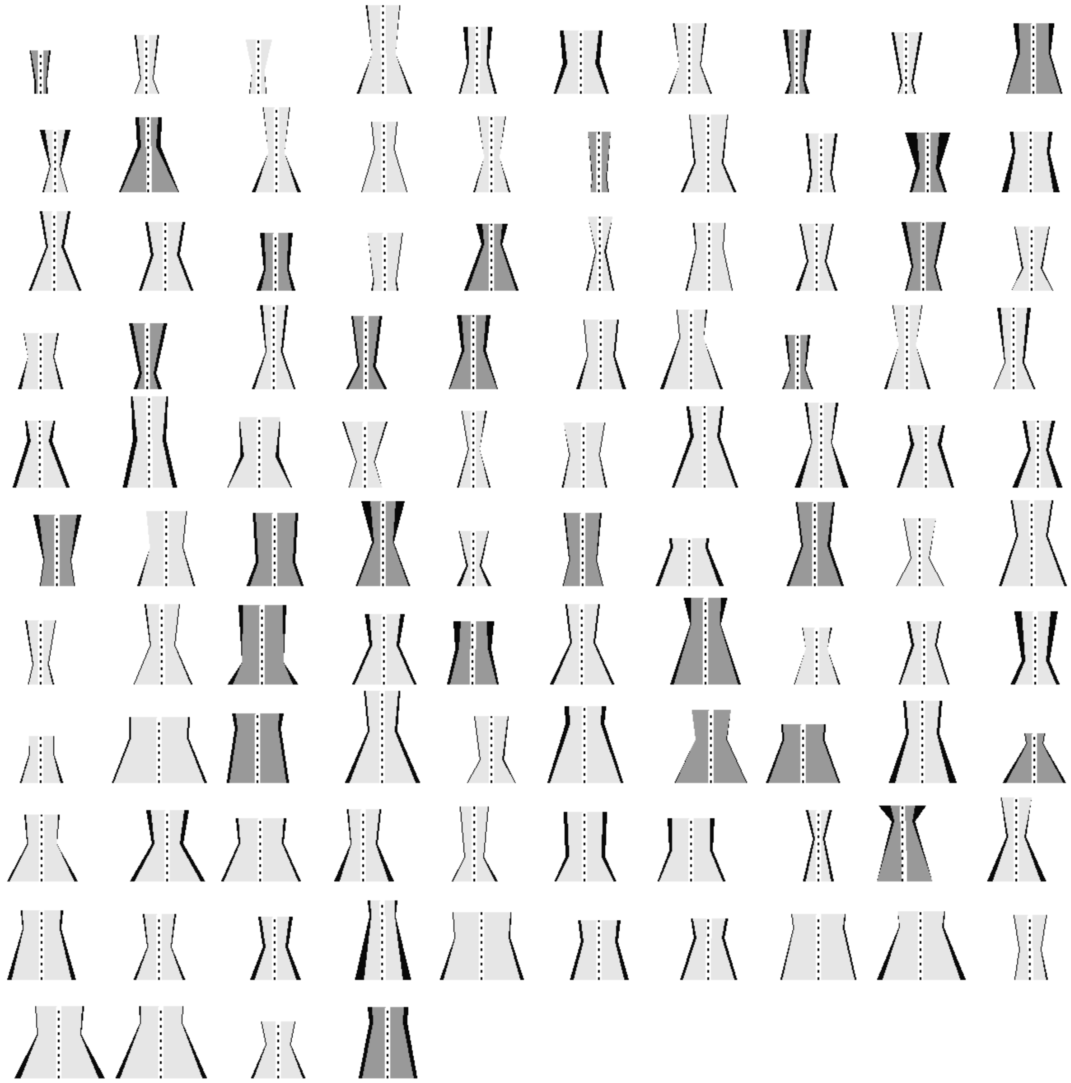

Supplementary figure 8: Schematic diagram representing all 104 holes by their height, base, minimal and top dimensions and ordered by preoperative visual acuity. The variability in the maxima and minima of the MA and BA is shown by the thickness of the borders of the holes. Holes with VMT are shaded in darker grey. The association between hole size and preoperative visual acuity is clearly seen with a trend towards bigger holes at the base of the diagram. The lack of association between hole size and VMT and preoperative visual acuity and VMT is also seen.
